# Surface Properties and Wear Resistance of Injectable and Computer-Aided Design/Computer Aided Manufacturing–Milled Resin Composite Thin Occlusal Veneers

**DOI:** 10.1055/s-0042-1750769

**Published:** 2022-10-11

**Authors:** Nesrine A. Elsahn, Hatem M. El-Damanhoury, Zainab Shirazi, Abdul Rahman M. Saleh

**Affiliations:** 1 Department of Clinical Sciences, College of Dentistry, Ajman University, Ajman, United Arab Emirates; 2 Center of Medical and Bio-allied Health Sciences Research, Ajman University, Ajman, United Arab Emirates; 3 Department of Operative Dentistry, Faculty of Dentistry, Cairo University, Cairo, Egypt; 4 Department of Preventive and Restorative Dentistry, College of Dental Medicine, University of Sharjah, Sharjah, United Arab Emirates

**Keywords:** CAD/CAM, hardness, injection molding, occlusal veneers, resin-based composite, roughness, wear

## Abstract

**Objectives** This study was conducted to investigate the microhardness, surface roughness (Ra), and wear behavior of thin occlusal veneers (TOV) fabricated from different injectable composite materials and compare them to a Computer-Aided Design (CAD)/Computer-Aided Manufacturing (CAM) resin-based material.

**Materials and Methods** A 1-mm occusal veneer preparation was done in a mandibular right second molar typodont tooth. The prepared model was duplicated to fabricate 32 replicas and divided into four groups ( *n* = 8). Standard TOV were fabricated either indirectly from Cerasmart blocks, Cerasmart, GC (CS), or directly from Beautifil Injectable X, Shofu (BF), G-ænial Universal injectable, GC (GU), or SonicFill 2, Kerr (SF) using the injection molding technique. All the specimens were subjected to both thermomechanical cyclic loading (TMC) in a chewing simulator. Wear measurement was conducted by three-dimensional (3D) scanning of the veneered models before and after TMC, and the difference in the volume of the sample was recorded as the volumetric material loss due to wear. Ra before and after TMC and Vickers microhardness (VHN) of the tested materials were measured using standardized samples ( *n* = 8). Representative samples from each group were investigated under a stereomicroscope and a scanning electron microscope.

**Statistical Analysis** One-way analysis of variance (ANOVA) was applied to detect the effect of material on VHN and wear. Two-way ANOVA was utilized to examine the impact of material and TMC on Ra. Multiple comparisons between the groups were conducted using Tukey's post hoc test ( *α* = 0.05). The Pearson's correlation coefficient was used to determine the relationship between hardness and wear and between roughness and wear ( *α* = 0.05).

**Results** CS exhibited the highest mean VHN ( *p* ≤ 0.001), followed by GU and SF which were statistically similar ( *p* = 0.883) but significantly higher than BF ( *p* < 0.001). After TMC, GU revealed the lowest Ra and volumetric wear (VW), followed by CS, BF, and SF ( *p* < 0.5). A highly significant correlation existed between Ra and VW ( *p* = 0.001, *R* ^2^ = 0.9803).

**Conclusion** The effect of TMC on the surface properties and wear resistance of the investigated TOV is material-dependent. GU injectable TOV are less influenced by TMC than CS milled TOV. In contrast, BF and SF demonstrated significant VW and Ra which might limit their clinical use as TOV.

## Introduction

Occlusal tooth wear is a current issue of concern caused by erosion, abrasion, attrition, or a combination of mechanical and chemical processes. 1 The clinical appearance of the lesion and the rate of its progression are affected by the etiological factors, patient's diet, habits, and occlusion. 2 Complications of tooth wear may involve a decrease in masticatory efficiency, loss of vertical dimension, hypersensitivity, and discoloration. 3 Early recognition, diagnosis, and treatment are of utmost importance to avoid lesion progression and eliminate the need for further complex treatment procedures, including treatment of hypersensitivity, occlusal rehabilitation, root canal treatment, and full-teeth coverage in severe cases. 4

Minimally invasive dentistry has recently become an area of interest. The durability and longevity of adhesive restorations are enhanced when maximum sound tooth structure is preserved. Traditional metal onlays and full-coverage crowns for the treatment of worn dentitions require more tooth structure removal compared with other conservative alternatives where composite and ceramic materials are utilized to fabricate ultrathin bonded posterior occlusal veneers. 4 5 6

Dental ceramics are known for their strength, high biocompatibility and survival rate, integrity, wear resistance, superior esthetic, and color stability. 7 Still, the abrasiveness of these materials against an enamel antagonist represents a clinical concern. 7 It has been postulated that polymer-based restorations can provide high fatigue resistance and may behave favorably in terms of intraoral repairability and opposing teeth preservation. 8 9 A recent prospective study reported acceptable long-term clinical results for indirect resin composite posterior restorations with 85% success rate at 9-year follow-up. 10

On the other hand, direct resin composite has been proposed as a cost-effective and less invasive alternative to indirectly fabricated restoration to restore chipped and worn dentition. The patient might prefer to choose direct composite veneers to avoid the high laboratory cost of indirect restorations and undergo a noninvasive treatment, particularly in countries where dental treatments are not under the insurance umbrella. 11 12 Many placement techniques for composite resin posterior veneers have been described. 13 14 Although the use of customized transparent polyvinylsiloxane guide or thermoplastic vacuum-formed matrix for constructing the semidirect composite veneers requires minor laboratory procedures and a second dental visit, it is a much easier alternative to free-hand composite build-up, especially when multiple teeth are involved. 15 In this technique, a transparent silicon index is customized for accurate and predictable duplication of a diagnostic wax-up using direct composite restorations without the need for tooth preparation. Both definitive and transitional restorations can be fabricated using this minimally invasive and cost-effective technique. 16

Owing to their low viscosity and good wettability, flowable composites exhibit better placement characteristics and marginal adaptation with fewer voids. 17 Their lower elastic modulus and stress absorbing capacity made them the material of choice for noncarious cervical lesions and for anterior veneers, where the restorations will be subjected to high compression forces induced by tooth flexure. 18 19 Flowable composites are preferred over packable composites for use with the transparent index technique due to their rheological properties that lead to accurate reproduction of the tooth morphology, in contrast to the relatively viscous packable composite. 16 17 20 However, the strength and wear resistance of the flowable composite in stress-bearing areas is questionable, even though both flowable and packable composites were found to show no statistical or clinical difference in any outcome as assessed in a recent meta-analysis. 18 19

In the last few years, a significant improvement in the mechanical properties and wear resistance of flowable composites was noticed. 21 Recently, a new group of injectable composites was introduced into the market. These composites combine the flowability and the strength needed to restore fractured and worn dentition and establish new vertical dimensions using the injection molding technique. 22 23

Wear can be defined as the progressive loss of substance resulting from mechanical interaction between two contacting surfaces. 24 The wear resistance, as well as the thermal and hydrolytic stability of resin composite materials, used to fabricate occlusal veneers are essential requirements for the durability of these restorations, as they will be in direct occlusal contact. Moreover, the challenging mechanical and/or chemical degradative oral conditions of patients in need of occlusal veneers remain a major clinical concern that requires careful selection of the resin composite material.

Neither the wear resistance of injectable thin occlusal veneers nor the surface properties of injectable universal composites were sufficiently examined in previous research. Therefore, this study was performed to investigate the surface roughness (Ra) and microhardness of injectable composites and their wear behavior as thin occlusal veneers under simulated oral environmental thermomechanical conditions compared with milled indirect resin-based restorations. The null hypotheses include the following: (1) no significant difference would be found concerning the microhardness, Ra, and wear behavior among the four materials used in this study for posterior occlusal veneers fabrication; and (2) thermomechanical cyclic loading (TMC) has no effect on the surface integrity and Ra of occlusal veneers fabricated from the four tested materials.

## Materials and Methods

The materials used in this study with their corresponding adhesives or cementing materials are listed in Table 1 .

**Table 1:** Materials used in the study with their corresponding adhesives/cementing materials

	Material	Composition	Filler composition	Filler Wt%/size	Lot no.	Manufacturer
Group 1	**Beautifil Injectable X (BF)** Injectable giomer, shade: A3	Bis-GMA, TEGDMA, Bis-MPEPP, polymerization initiator, pigments, others	S-PRG fillers based on aluminofluoro-borosilicate glass, Al2O3	64% 0.8 μm	111901	Shofu Inc., Kyoto, Japan
	**BeautiBond** Self-etching one component dental adhesive	Acetone, distilled water, bis-GMA, Carboxylic acid monomer, TEGDMA, phosphonic acid monomer, others			091927	
Group 2	**G-ænial Universal injectable (GU)** Injectable composite, shade: A3	UDMA, bis-EMA, methacrylate monomers, photoinitiator, UV-light absorber, pigments	Silica Barium glass	69% 150 nm	1904041	GC Corp., Tokyo, Japan
	**G-Premio Bond** one component light-cured adhesive	4-MET, phosphate monomer, thiophosphate monomer, dimethacrylate, acetone, water, photoinitiator			2001281	
Group 3	**SonicFill 2 (SF)** Bulk-fill composite, shade: A3	Bis-GMA, TEGDMA, Bis-EMA	Silica, barium glass, YbF3, mixed oxides	83.5% 0.4 μm	7207987	Kerr Corp., Orange, California, United States
	**OptiBond Universal** Single component universal adhesive	HEMA, GDMA, GPDM, acetone, ethanol			7365205	
Group 4	**Cerasmart (CS)** Resin matrix ceramic, shade: A3 HT	Bis-MEPP, UDMA, dimethacrylate	Silica Barium glass	71% <500 nm	1908231	GC Corp., Tokyo, Japan
	**Ceramic Primer II** Primer for ceramic and composite bonding	Silane, phosphate monomer, methacrylate, ethanol			2009151	
	**G-CEM LinkForce** Adhesive resin cement	Paste A: bis-GMA, UDMA, DMA, initiator, pigments Paste B: bis-MEPP, UDMA, DMA, initiator, bis-EMA, dibenzoyl peroxide, BHT			1912201	

Abbreviations: 4-MET, 4-methacryloyloxyethyl trimellitic acid; BHT, butylated hydroxytoluene; bis-GMA, bisphenol-A-glycidyldimethacrylate; Bis-MEPP, 2,2-bis (4-methacryloxyphenyl) propane; bis-MPEPP, 2,2-bis-(4-methacryloxy polyethoxy) phenyl]propane; DMA, dimethacrylate; GDMA, glycidyl dimethacrylate; GPDM, glycerophosphate dimethacrylate; HEMA, hydroxy ethyl methacrylate; HT, high translucency; bis-EMA, bisphenol A ethoxylated dimethacrylate; S-PRG, surface prereacted glass-ionomer; TEGDMA, triethyleneglycol dimethacrylate; UDMA, urethane dimethacrylate; UV, ultraviolet.

Note: Other symbols according to the elements of the periodic table.

### Specimen Fabrication

A mandibular right second molar typodont tooth (Nissin Dental Products Inc.) was scanned using a three-dimensional (3D) scanner (CEREC inLab 3D XL CAD System, Dentsply Sirona), and the scan was saved as a full-contour reference model to serve as a reference for the occlusal veneer preparation and fabrication.

A conservative standardized occlusal veneer preparation of 1 mm was prepared by an experienced restorative specialist under magnification. The prepared tooth was then duplicated to fabricate 32 replicas from cold-curing orthodontic acrylic resin (Vertex Orthoplast, Vertex-Dental, Soesterberg, the Netherlands). The acrylic resin replicas were then randomly assigned to four different groups ( *n* = 8) according to the material used to fabricate the occlusal veneers, as listed in Table 1 .

### Fabrication of the Transparent Silicon Index

The scanned unprepared typodont tooth was placed in the lower typodont model with all other teeth in place. A transparent silicone impression material (Exaclear, GC Corp., Tokyo, Japan) was loaded in a nonperforated stock tray and seated over the model centered on the mandibular right second molar. After setting, the loaded tray was removed, and the transparent index was carefully separated from the tray.

### Pretreatment of Bonded Surfaces

Prior to the cementation of each indirect veneer or the injection of each direct veneer, the occlusal surfaces of the acrylic-resin tooth replicas were sandblasted for 10 seconds with 50-μm Al _2_ O _3_ at a pressure of 2 bars and a source to sample distance of 2 cm, they were then cleaned in an ultrasonic water bath and dried.

### Fabrication of Direct Occlusal Veneers

Two holes were created in the transparent silicon index using a syringe tip from inward to outward at the occlusal surface of tooth no. 47, one hole for the injection of the material and the other to act as a vent. For SF group, slightly larger holes were created in the transparent silicon index using a cylindrical high-speed diamond bur with a rounded edge (no.: 837LKR.314, Komet, Germany) in the same locations to allow insertion of the tip of the cartridge. The corresponding bonding agent that is recommended by the manufacturer of each direct material was applied to the sandblasted surfaces of the specimens and light cured for 10 seconds. The silicon index was seated precisely over the model, and each injectable composite material was injected and light cured from the occlusal, buccal, and lingual surfaces. Each surface was cured for 10 seconds, following the manufacturers' instructions. The transparent index was then removed, and additional light curing was done from each surface for 10 seconds. Finishing and polishing was performed using a two-step composite finishing and polishing set (no.: 4546.000, Komet, Germany)

The light curing in this study was performed using an LED light-curing unit (LCU) (Bluephase N, Ivoclar Vivadent; United States) with an output power of 1,200 mW/cm ^2^ . A hand-held radiometer (Curing Radiometer, Demetron, Danbury, Connecticut, United States) was used to check the power density of the LCU periodically.

### Fabrication and Cementation of Indirect Occlusal Veneers

Each resin model was scanned with a tabletop 3D digital scanner (inEos X, Sirona, Bensheim, Germany) and an occlusal veneer was designed using a Computer-Aided Design (CAD) software (inLab SW 4.2, Sirona, Bensheim, Germany), using the scanned image of the unprepared tooth as a reference. The veneers were then milled in a milling machine (Cerec MC XL, Sirona, Bensheim, Germany) from resin-based hybrid CAD/Computer-Aided Manufacturing (CAM) blocks (CeraSmart, GC Dental).

The fitting surfaces of the veneers were sandblasted for 10 seconds using 50 μm particles of Al _2_ O _3_ at a pressure of 2 bars and a source to sample distance of 2 cm, washed with an air/water spray, and then dried. A silane-based primer (Ceramic Primer II) was applied to the fitting surface and left to react for 60 seconds, then dried with air. A dual-cure adhesive resin cement (G-CEM LinkForce, GC Dental) was used for cementation. The veneers were seated and a standardized load of 50 g was applied on the occlusal surface for 30 seconds. Excess cement was removed using a microbrush, and the cement was light cured for 20 seconds from the occlusal aspect. Then the margins of the veneers were covered with a glycerin-based gel (Oxigaurd, Kuraray Inc., Japan) and light curing was performed at the margins for 20 seconds. Afterward, finishing and polishing were performed as mentioned earlier.

### Thermomechanical Cyclic Loading

Eight specimens from each group were mounted in an eight-chamber chewing simulator (CS-8, SD Mechatronik, Germany) for TMC. A Steatite ceramic ball with a 6-mm diameter was used as the antagonist to strike the buccal cusps of the veneers. A 5-kg vertical load was used with a 6-mm vertical movement and 2-mm horizontal sliding in each masticatory cycle. Each group was loaded for 500,000 chewing cycles and was simultaneously subjected to 10,000 thermal cycles between 5 and 55°C.

### Volumetric Wear Analysis

**Fig. 1:**
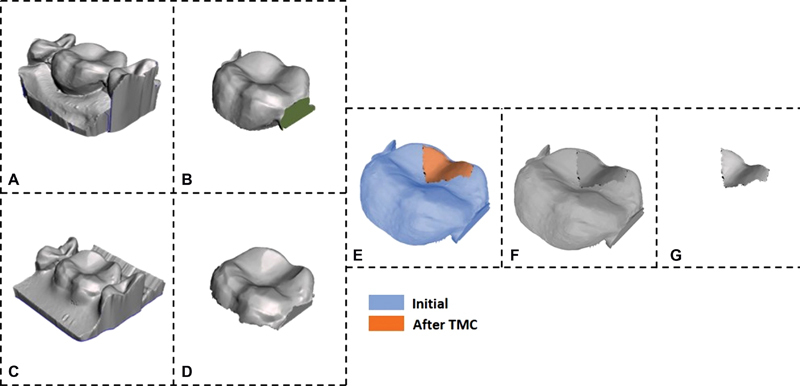
Volumetric wear measurement of occlusal veneers. ( **A** ) Initial mesh; ( **B** ) Initial mesh fixed; ( **C** ) Mesh after TMC; ( **D** ) Mesh after TMC fixed; ( **E** ) Alignment step; ( **F** ) Difference mesh; ( **G** ) Final mesh. The initial ( **A** ) and after TMC ( **C** ) meshes were imported to Autodesk Meshmixer (Version 3.5.474) to fix mesh by flipping normals, closing holes, and removing overlapping triangles. A plane cut was applied to reduce the volume of the meshes as in ( **B** ) and ( **D** ). Then, the meshes were aligned ( **E** ) in Meshlab (Version 2021.10) and a difference Boolean operation was applied. Finally, the mesh was cleaned and trimmed to the volume of interest. TMC, thermomechanical cyclic loading.

The 3D wear analysis was done by scanning the 32 veneered models before and after TMC with the previously mentioned tabletop 3D digital scanner. The volumetric loss was calculated by the superimposition of the 3D models before and after TMC and a subtraction process using a 3D image processing software Meshmixer (Autodesk, California, United States) and MeshLab (Consiglio Nazionale delle Ricerche, National Research Council, Rome Italy,). The difference in the volume before and after TMC was recorded as the volumetric material loss due to wear ( Fig. 1 ). Representative images of the wear pattern of each group were taken using a stereomicroscope with a built-in camera (Leica Wild M420, Leica, Bensheim, Germany) at ×40 magnification.

### Scanning Electron Microscope Analysis

Three representative samples from each group were sputter-coated with 100 Å Gold-Palladium (EMS 7620 Mini Sputter Coater, Hatfield, Pennsylvania, United States). Micromorphological analysis was performed under different magnifications up to ×5,000, using a scanning electron microscope (SEM; VEGA3 XM–TESCAN, Kohoutovice, Czech Republic) operating at 10 kV acceleration voltage and 15 ± 1 mm working distance.

### Sample Preparation for Microhardness and Surface Roughness Testing

Standardized molds with 2-mm thickness and 5-mm internal diameter were utilized to fabricate disc-shape samples from Beautifil Injectable X (BF), G-ænial Universal injectable (GU), and SonicFill 2 (SF) (n=16). The materials were packed in the model between two transparent mylar matrix strips (Universal Strip, DML, Germany) and glass slides then a 500 g weight was applied for 20 seconds. Subsequently, the weight and the glass slide on top were removed then the resin composite cylinders were light cured from the top side only for 10 seconds. The top-side mylar strip was removed, and the material was light cured for another 10 seconds. The cured composite cylinders were carefully pulled out from the mold, and the bottom surface of the specimens was identified with an indelible mark. For the Cerasmart (CS) group, 2-mm slices were sectioned using a precision saw (IsoMet 1000, Buehler, Germany). The top surfaces of all the specimens were polished with Sof-Lex abrasive discs (coarse, medium, fine, superfine; 3M-ESPE Dental Products, St. Paul, Minnesota, United States). Afterward, the specimens were kept in an incubator for 24 hours at 37°C.

### Vickers Microhardness Testing

Eight samples from each group were randomly assigned to the microhardness test by applying a 300-g load and a dwell time of 15 seconds. The testing was performed utilizing a Vickers microhardness (VHN) tester (FM-800, Future-Tech Corp., Japan), following the ISO standards for composite resins developed in conjunction with the ADA. The computer software (Hardness-Course Vickers/Brinell/Rockwell copy correct IBS 2012 edition 10.4.4) automatically calculated the Vickers hardness number. The average of three sequential measurements was taken for each sample.

### Surface Roughness Testing

The Ra was measured utilizing a surface finish gage roughness tester (Mitutoyo suftest-211 surface roughness tester, Mitutoyo Corporation, Tokyo, Japan) with a 2-μm diamond indenter running at 0.5 mm/s. The cut-off value for Ra was 0.25 mm, and the traversing distance of the stylus was 1.25 mm. The measurements were done on the top surfaces of the remaining eight samples from each group. Three different measurements were taken from each sample, and the average Ra of the three readings was then calculated.

### Statistical Analysis

The data collected were analyzed using SPSS software (SPSS version 26.0, IBM Corp., Armonk, New York, United States). Test of normality was conducted using Shapiro–Wilk test with a 0.05 significance level. One-way analysis of variance (ANOVA) was utilized to determine the effect of material on the VHN and volumetric wear (VW). The effect of material and TMC on Ra of samples was analyzed with two-way ANOVA. Tukey's post hoc test (LSD) was used for multiple comparisons between the groups. The significance level was set at *p* ≤ 0.05 with confidence level (95%). The Pearson's correlation coefficient was used to determine the relationship between hardness and wear and between roughness and wear ( *α* =0.05).

## Results

Mean VHN and VW of all tested materials are presented in Table 2 , and mean values of Ra before and after TMC are shown in Table 3 .

**Table 2:** Mean (standard deviation) Vickers microhardness (VHN) and volumetric wear of the tested groups.

Material	VHN	Volumetric wear (mm ^3^ )
Beautifil Injectable X	34.45 (3.88) ^a^	5.8 (0.75) ^a^
G-ænial Universal injectable	46.42 (3.54) ^b^	1.9 (0.26) ^b^
SonicFill	46.78 (4.23) ^b^	10.4 (1.44) ^c^
Cerasmart	55.41 (4.99) ^c^	3.7 (1.12) ^d^

Note: Within each column, values with the same superscript letter are statistically similar ( *p* > 0.05).

**Table 3:** Mean (standard deviation) surface roughness (Ra) of the tested groups before and after thermomechanical cyclic loading (TMC)

Material	Ra before TMC (μm)	Ra after TMC (μm)
Beautifil Injectable X	0.065 (0.0177) ^a^	1.934 (0.223) ^b^
G-ænial Universal injectable	0.051 (0.013) ^a^	1.036 (0.348) ^c^
SonicFill	0.057 (0.011) ^a^	3.765 (1.082) ^d^
Cerasmart	0.061 (0.015) ^a^	1.518 (0.582) ^e^

Note: Within each column and each row, values with the same superscript letter are statistically similar ( *p* > 0.05)

One-way ANOVA revealed that the type of material has a significant effect on VHN and VW ( *p* < 0.001). VHN of CS was significantly higher than that of SF, GU, and BF ( *p* ≤ 0.001). However, the difference between VHN of SF and GU was not statistically significant ( *p* = 0.883). On the other hand, VHN of BF was significantly lower than the other tested groups ( *p* < 0.001). Furthermore, statistical analysis indicated that GU scored a significantly lower VW than CS ( *p* = 0.003). Meanwhile, the differences in VW among the other tested groups were highly significant ( *p* < 0.001).

**Fig. 2:**
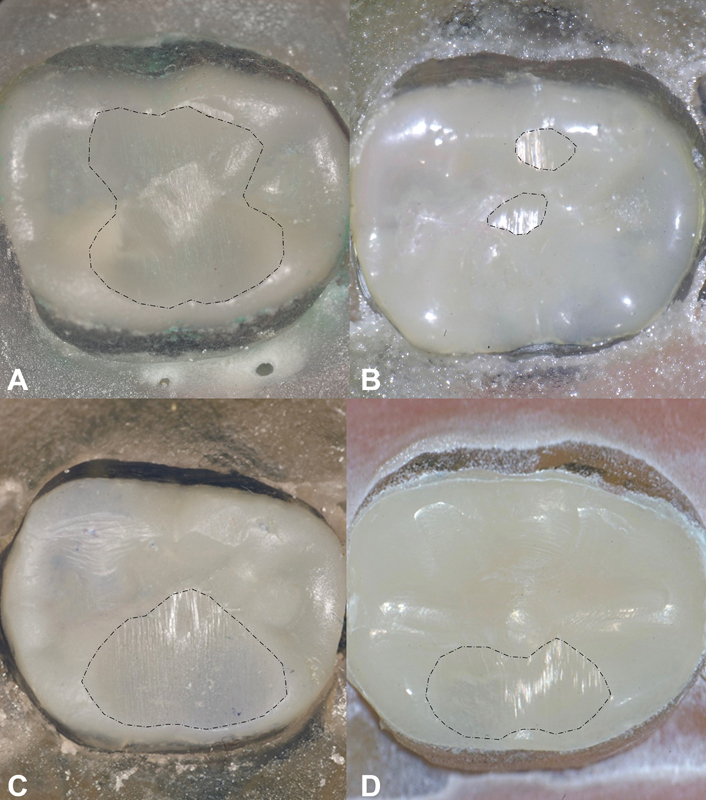
Maximum wear patterns of the tested groups. The dotted lines mark the borders of wear facets. ( **A** ) BF group with a wide wear patch that has a medium depth. ( **B** ) GU group with two isolated wear patches with minimal width and depth. ( **C** ) SF group with a deep and extensive wear facet mesiodistally. ( **D** ) CS group with shallower and less extensive wear facets than SF and BF groups. BF, Beautifil Injectable X; CS, Cerasmart; GU, G-ænial Universal injectable; SF, SonicFill 2.

**Fig. 3:**
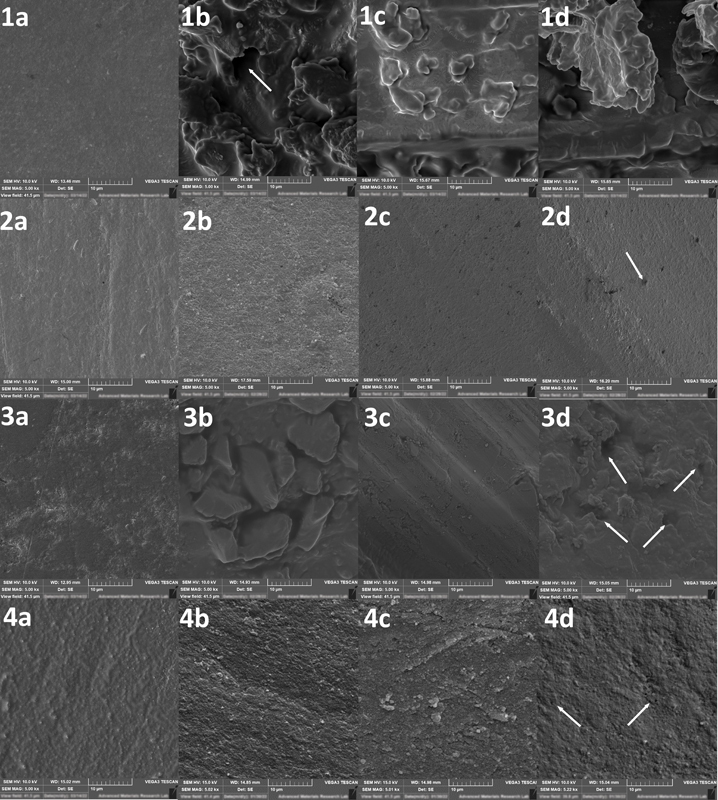
Representative SEM microimages for the tested groups under ×2,000 and ×5,000 magnifications. 1: BF, 2: GU, 3: SF, 4: CS, a: polished samples, b: surface subjected to thermocycling only (TC); c and d: surfaces subjected to thermomechanical cyclic loading (TMC). Thermocycled samples (1–4b) showed generalized increase in surface roughness in comparison to polished groups (1–4a). Thermocycling altered the surface topography of BF significantly (1b) and exposed large and irregular fillers of SF (3b) compared with small and uniform fillers of GU (2b) and CS (4b). TMC left marked deep striation on wear facets' surfaces of BF (1c) and SF (3c). Shallower striations found in GU (2C, 2d) and CS (4c, 4d) groups. White arrows point to empty spaces representing eluted fillers. BF, Beautifil Injectable X; CS, Cerasmart; GU, G-ænial Universal injectable; SEM, scanning electron microscope; SF, SonicFill 2.

Two-way ANOVA revealed that TMC significantly increased Ra of the four TOV materials under investigation ( *p* < 0.001). While no statistically significant difference existed among the tested groups in terms of the initial Ra ( *p* > 0.05), following TMC, Ra of SF (3.76 ± 1.08) was significantly higher than BF (1.93 ± 0.22), CS (1.51 ± 0.58), and GU (1.04 ± 0.35) ( *p* < 0.001). The differences between Ra of the four tested groups after TMC were statistically significant ( *p* < 0.05). Stereomicroscope images of the wear patterns after TMC are displayed in Fig. 2 . The micromorphology of representative polished, thermocycled (TC), and TMC surfaces from each material are demonstrated in Fig. 3 .

There was no statistically significant correlation between wear and hardness of the tested materials ( *p* = 0.281, *R* ^2^ = 0.0367), while there was a highly significant correlation between roughness and wear ( *p* = 0.001, *R* ^2^ = 0.9803).

## Discussion

In this study, the surface properties of direct occlusal veneers, made of injectable resin-based composites (RBCs) (GU and BF) and a sonic-activated bulk-fill composite (SF), were compared with CAD/CAM milled prepolymerized resin-based occlusal veneers (CS). The statistical analysis revealed that the material type significantly affected the VHN of polished samples, VW, and Ra after TMC ( *p* < 0.05). Therefore, the first hypothesis was rejected. On the other hand, TMC dramatically increased the Ra of the occlusal veneers ( *p* < 0.001), and all the tested materials showed variable degrees of loss of structural integrity after TMC. However, SF and BF occlusal veneers exhibited more significant VW than GU and CS. Consequently, the second hypothesis was also rejected.

Hardness is defined as the resistance of a material to permanent indentation. 25 Studies have linked the filler fraction of resin composites to their surface hardness, compressive strength, and stiffness. 25 26 27 Elzoheiry et al emphasized the strong relationship between filler particles, the link between polymer matrix and filler particles, and surface hardness. 28 The findings of our study revealed a higher VHN for CS than GU and SF. However, BF exhibited the lowest VHN. The filler loading of CS, SF, GU, and BF are around 71, 83, 69 and 64 wt%, respectively. The difference in VHN between CS, GU, and BF can be attributed to their filler type and fraction. BF is a flowable giomer-based composite with prereacted aluminofluoro-borosilicate glass fillers (S-PRG). On the other hand, CS and GU contain barium glass and silica nanoparticles. The incorporation of S-PRG fillers in BF could negatively influence its VHN. A previous investigation reported a significantly softer surface for giomer-based composites than more heavily loaded RBCs with smaller filler particles. 29 Although the filler loading of SF is higher than that of CS, CS showed significantly higher VHN ( *p* < 0.05). CS is a prepolymerized material, and its manufacturing technique might have improved the material properties, particularly the degree of conversion and the filler-matrix binding.

The roughness of RBCs is dependent on many factors, such as the particle size of the fillers and its effect on the percentage of surface area occupied by filler particles, the degree of polymer conversion, the interaction between fillers and matrix, as well as hardness. 30 While no significant difference was found between the initial Ra of polished discs from different material groups ( *p* > 0.05), SF exhibited the highest Ra, and VW followed by BF, CS, then GU after TMC. The difference between the four tested groups in terms of their Ra and VW following TMC was statistically significant ( *p* < 0.05).

Wear resistance of RBCs is strongly influenced by the type, shape, and size of the fillers, in addition to the inter-filler spacing. Stawarczyk et al reported that an increase in filler loading and a decrease in filler particle size enhanced the wear resistance. 31 Furthermore, a group of intrinsic factors, such as the degree of polymerization, hydrolytic degradation, water absorption, fatigue, elastic modulus, hardness, flexural strength, and surface finish was found to have a significant effect on restorative materials wear. 32 Materials with high initial surface polish and hardness are expected to wear less clinically. However, it was proved that the recorded properties of most restorative materials could be altered after short- or long-term exposure to various deteriorating oral environmental factors, depending on the material's inherent structure. 33 Therefore, simulating the combined effect of thermal fluctuation and load cycling in an aqueous environment during testing was respected in this study following the previous recommendations. 34 The increase in Ra of the wear facets' surfaces of the four groups after TMC, as illustrated in Table 3 and Fig. 3 , and the considerable variation in Ra and VW between the tested materials after TMC demonstrates that the resistance to hydrolytic degradation, thermal fatigue, and cyclic loading was material dependent. Additionally, VHN solely cannot give an accurate prediction for the wear resistance of materials.

Liquid uptake and thermal fatigue can lower the wear resistance of RBCs, as it employs a degradative effect on their resin matrix and filler component. 34 Diffusion of water can leach unreacted monomers from the resin matrix. Simultaneously, thermal fatigue has a plasticizing effect on the resin matrix and may cause degradation of the organic silane coating, promoting the dislodgement and elution of filler particles. Moreover, water sorption and thermal decomposition can separate the chains, interrupt the arrangement of the polymer chains in the polymerized network, eliminate side groups, and lead to oxidation of the polymer. 35

According to Bucuta and Ilie, the ability of SF to transmit sufficient light up to 5-mm increment thickness was attributed to the presence of considerably irregular-shaped large-size fillers which contribute to improving the polymerization efficiency. 26 On the other hand, the shape and size of the fillers negatively influenced the material Ra and its resistance to wear under the challenging degradative effect of TMC.

The SEM observations of SF group ( Fig. 3b–d ) after TMC demonstrated considerably large irregular glass filler particles, with noticeable plucking of glass from the SF surface and fracturing of the larger glass filler particles. Our findings are consistent with other studies which reported significantly greater volumetric loss with deeper localized and generalized wear facets of SF groups than the other packable and high viscosity bulk-fill resin composites investigated. 26 36

Similarly, the significantly greater material loss and Ra of BF after TMC can be attributed to its lower VHN and larger average particle sizes (0.8 μm), as compared with 500- and 150-nm fillers found in CS and GU groups, respectively. The negative correlation between roughness and hardness of a giomer-based material reported in a previous study can support our findings. 29 37 38 BF's low wear resistance can also be attributed to its higher tendency for hydrolytic degradation and thermal decomposition owing to the combination of bis-GMA and TEGDMA in its resin matrix which have high water sorption around 33.5 and 69.5 μg/mm ^3^ , respectively, with the bioactive fluoride-releasing S-PRG fillers that have a well-known affinity to water absorption and solubility. 37 38 39

Conversely, GU matrix is based on UDMA and bis-EMA monomers which are characterized by lower water sorption of 29.5 and 20.10 μg/mm ^3^ , respectively. While the organic component of CS is based on a mixture of Bis-MEPP and UDMA monomers.

The dispersed nanosized barium particles (150 nm) in GU, which are firmly bonded into the resin matrix through full-coverage Silane Coating (FSC) technology, might have guaranteed a solid and stable filler-matrix bond that can significantly resist the thermomechanical aging and might also justify the significant increase in VHN of GU compared with BF. 22 Additionally, the inclusion of bis-EMA in GU might reduce water sorption and hinder subsequent material decomposition. These findings emphasize that the impact of filler–matrix binding, type of matrix monomers, and fillers composition might play a more significant role in the degradation and wear resistance of RBCs than their filler fraction.

The main objective of the current study was to assess VW of TOV after fatigue loading of 500,000 chewing cycles which corresponds approximately to 2 years of clinical service. 40 The findings of this study can assist clinicians in selecting the best wear-resisting resin-based material for TOV. However, future work shall investigate the long-term clinical performance of injectable TOV, their fracture strength, fatigue resistance, and survival rate after different chemical and mechanical challenges.

## Conclusion

The following inferences can be drawn as conclusion:

- The effect of the stimulated aging by TMC on the surface integrity and roughness of the materials under investigation is material dependent.
- A strong link was found between the wear resistance of RBCs and their surface roughness after TMC.
- GU directly injected thin occlusal veneers are more durable than CS milled alternatives. Conversely, SF and BF materials are not recommended as occlusal veneers owing to their significant degradation under TMC.
